# Dye-coupling between neonatal spinal motoneurons and interneurons revealed by prolonged back-filling of a ventral root with a low molecular weight tracer in the mouse

**DOI:** 10.1038/s41598-019-39881-0

**Published:** 2019-03-01

**Authors:** Dvir Blivis, Melanie Falgairolle, Michael J. O’Donovan

**Affiliations:** Developmental Neurobiology Section, National Institute of Neurological Disorders and Stroke, National Institutes of Health, Bethesda, 20892 MD USA

## Abstract

We investigated dye-coupling between motoneurons in the L6 segment of the neonatal mouse spinal cord that contains limb-innervating motoneurons and sexually dimorphic motor nuclei. Using an isolated spinal cord preparation, we back-filled the cut, L6 ventral root with the small molecule Neurobiotin, or the much larger dextran-conjugated fluorophores for 16–24 hours. Motoneurons and parasympathetic preganglionic neurons were filled with both markers, but dye-coupling was only seen with Neurobiotin fills. Following a neurobiotin fill, fluorescence was observed in contralateral motoneurons, in motoneurons innervating adjacent ventral roots, and in ChAT-negative, putative interneurons outside of the motoneuron pools in addition to the directly back-labeled L6 motoneurons. It is known that the gap junction protein connexin-36 is widely expressed in the sexually dimorphic motoneurons of the L6 segment, suggesting that the dye-coupling is mediated by gap junctions. The technique has revealed previously unknown connections of motoneurons in the neonatal mouse cord that are likely to play important roles in the development and function of spinal circuits.

## Introduction

Gap junctions between neurons are prominent in the developing nervous system^1,2^ and serve important functions in the adult nervous system - for review see^3^. Traditionally, gap junctional coupling between neurons has been investigated using intracellular recordings from pairs of individual neurons^4^. Alternatively, the existence of gap junction coupling can be inferred by showing that an intracellularly injected, low molecular weight dye can label cells other than the one that was injected^5,6^. A major limitation of both approaches is that they require intracellular recording from individual neurons which greatly limits the number of cells that can be investigated. An alternative strategy is to label a population of neurons with a small molecular weight dye that can traverse gap junctions and label the coupled neurons. This approach was initially used to label neostriatal neurons following injection into the prefrontal cortex of rats^7^. The first report using the method with motoneurons revealed a dye-coupled presynaptic network projecting to the sonic motoneurons of a Teleost fish^8^. Since then, the technique has been used to examine population dye-coupling in the turtle^9^ and the frog^10,11^. Surprisingly, however, the method has not been used to investigate dye-coupling in mammals. Here, we used the method to examine dye-coupling of motoneurons in the L6 segment of the neonatal mouse spinal cord. We focused on the L6 segment because work in the adult rat, using intracellular injections of Neurobiotin into single motoneurons in fixed tissue, had demonstrated dye coupling between sexually dimorphic motoneurons and other neurons^12^. However, such coupling has not otherwise been reported, and it is unclear what the effect of fixation is upon dye-coupling. Nevertheless, our work confirmed the existence of coupling between ipsilateral motoneurons and revealed coupling between ipsi- and contralateral motoneurons, and between putative interneurons and ispilateral motoneurons in the L6 segments thereby demonstrating the feasibility of the approach in the mammalian spinal cord. Some of this work appeared in abstract form^13^.

## Results

**Figure 1:**
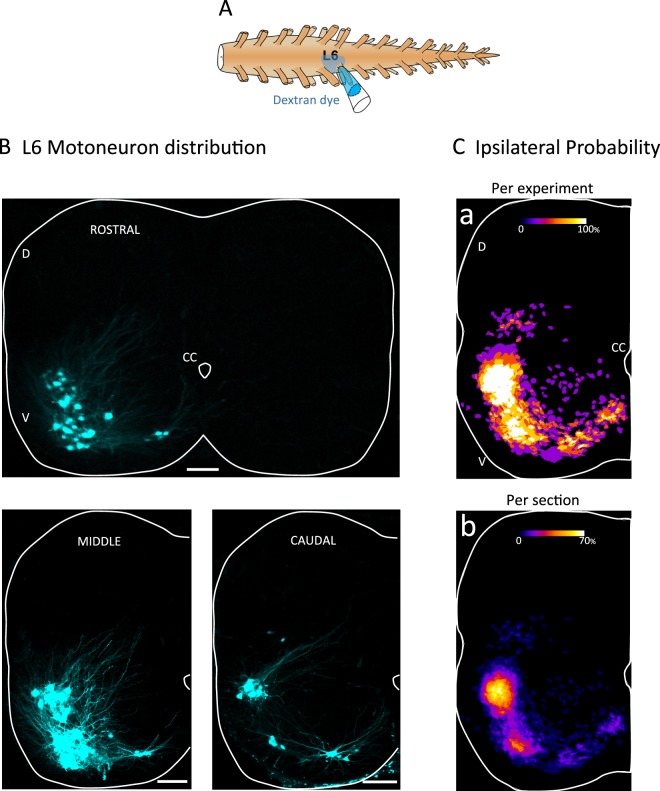
Distribution of L6 neurons labeled by backfilling the L6 ventral root with a high molecular weight dye. (**A**) Experimental arrangement for neurons in L6 segments with the high molecular weight dye Cascade Blue Dextran (10,000 MW). (**B**) Spatial distribution of labeled neurons at different rostrocaudal levels (ROSTRAL, MIDDLE, CAUDAL) of the L6 segment. Neurons were never detected on the contralateral side of the fill (example in the ROSTRAL section). (**C**) Map of the probability of finding labeled neurons per cord (a; n = 4) or per section (b; n = 41, 4 cords). Calibration bar 100 µm. V- Ventral. D- Dorsal. CC- Central Canal.

In the first set of experiments (n = 4), we characterized the location of motoneurons in the L6 segment of the neonatal mouse spinal cord. To achieve this, the cut L6 ventral root was back-filled overnight with high molecular weight dextran dye that does not cross gap junctions (Fig. 1A).

Figure 1B shows the spatial organization of the backfilled neuron pools along the rostrocaudal axis of the segment. In the rostral part of the segment, neurons (likely motoneurons rather than parasympathetic preganglionic neurons due to their location) are concentrated into two groups: a lateral and a smaller medial group. In the middle of the segment, the number of motoneurons increases and the lateral group is segregated into a dorsal and a ventral component. At the caudal end of the segment three distinct groups are evident. The probability of finding parasympathetic preganglionic neurons is rather low (the most dorsal group of neurons, Fig. 1Ca,b) because they are present only in the very caudal part of the L6 segment. No neurons were detected contralateral to the dextran fill. The location of the labeled motoneurons was quantified (Fig. 1C) by determining the probability of occurrence of a labeled cell at each pixel of the image (see methods). This analysis revealed that L6 backfilled neurons (motoneurons and parasympathetic preganglionic neurons) are concentrated in the lateral and ventromedial part of the segment (Fig. 1Ca- average analysis per experiment n = 4; 1Cb- average analysis per 100 µm section; n = 41 sections). Infrequently, some cells were also detected in the ipsilateral intermediate zone (laminae VII).

**Figure 2:**
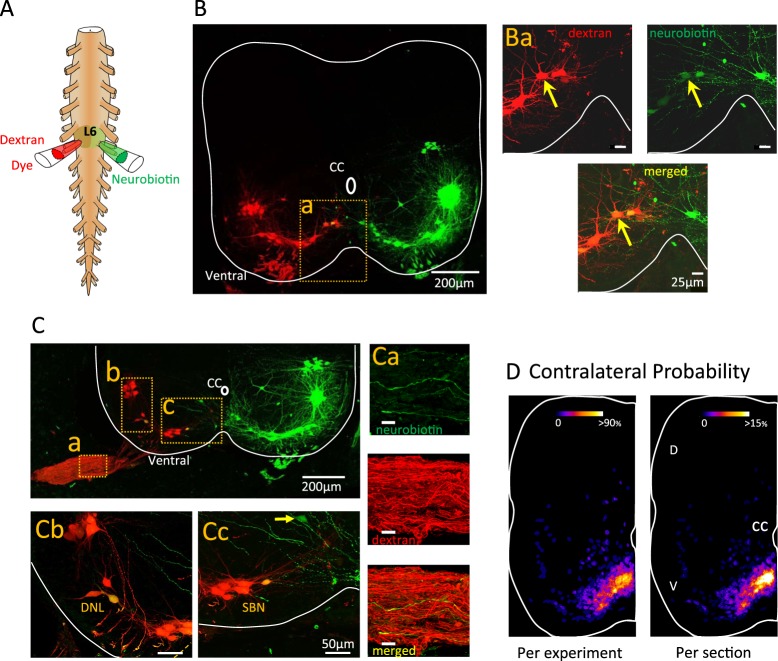
L6 backfilled neurons are dye coupled to some contralateral motoneurons. (**A**) Experimental arrangement for back-filling. The left L6 ventral root was retrogradely loaded with a low molecular weight dye (Neurobiotin; green) while the contralateral ventral root was retrogradely filled with a high molecular weight dye (Texas Red Dextran; red). (**B**) A z-stack projection (65 µm; 34 optical sections at 1.98 µm each) micrograph of a transverse section of the L6 segment showing neurons loaded with low (green) and high (red) molecular weight dyes. (Ba) While the Texas Red Dextran does not label contralateral neurons, the Neurobiotin does. The yellow arrow identifies a dye-coupled contralateral motoneuron. (**C**) Dye-coupled neurons can be found within different motoneurons pools (Cb – Dorsolateral nucleus (DNL); Cc – Dorsomedial nucleus (DMN)). The yellow arrow identifies a contralaterally dye-coupled neuron that was not labeled by the dextran fill. (Ca) Some axons in the dextran backfilled ventral root (in red) were labeled with Neurobiotin (green) confirming their identity as motoneurons. (**D**) Probability analysis of the dye-coupled neurons per experiment (see text for details). V- Ventral. D- Dorsal. CC- central canal.

In the next set of experiments, we performed fills of the right and left L6 ventral roots to compare the labeling pattern of neurons backfilled with either Neurobiotin or a dextran-conjugated fluorophore. Neurobiotin is a low molecular weight dye that has been shown to cross gap junctions^6^ whereas the dextran-conjugated dye does not. An example of this type of experiment is shown in Figure 2. The dextran fill only labeled ipsilateral neurons. By contrast, neurons labeled with Neurobiotin were observed contralateral to the filled root (Fig. 2Ba; 2Cb-Cc), in the ventrolateral (2Cb), ventromedial (2Ba; 2Cc) and intermediate areas of the cord. Some of the contralaterally labeled neurons were also labeled from the dextran backfill, indicating that they were probably motoneurons (Fig. 2Bb; 2Cb; 2Cc). Consistent with this interpretation, we observed some Neurobiotin-labeled axons in the contralateral ventral root (Fig. 2Ca).

We quantified the probability of detecting contralaterally labeled neurons as described for the data in Figure 1. Figure 2D shows that most of the dye-labeled, contralateral neurons (n = 701 neurons in 15 cords) were found in the ventromedial part of the cord (2D left - Per experiment, n = 15 cords; 2D right - per section, n = 172 sections from 15 cords). The number of contralaterally labeled cells ranged from 6–109 in all experiments with and average number per experiment of 46.6 ± SD 28.4. The average number per section was 4.4 ± SD 3.

**Figure 3:**
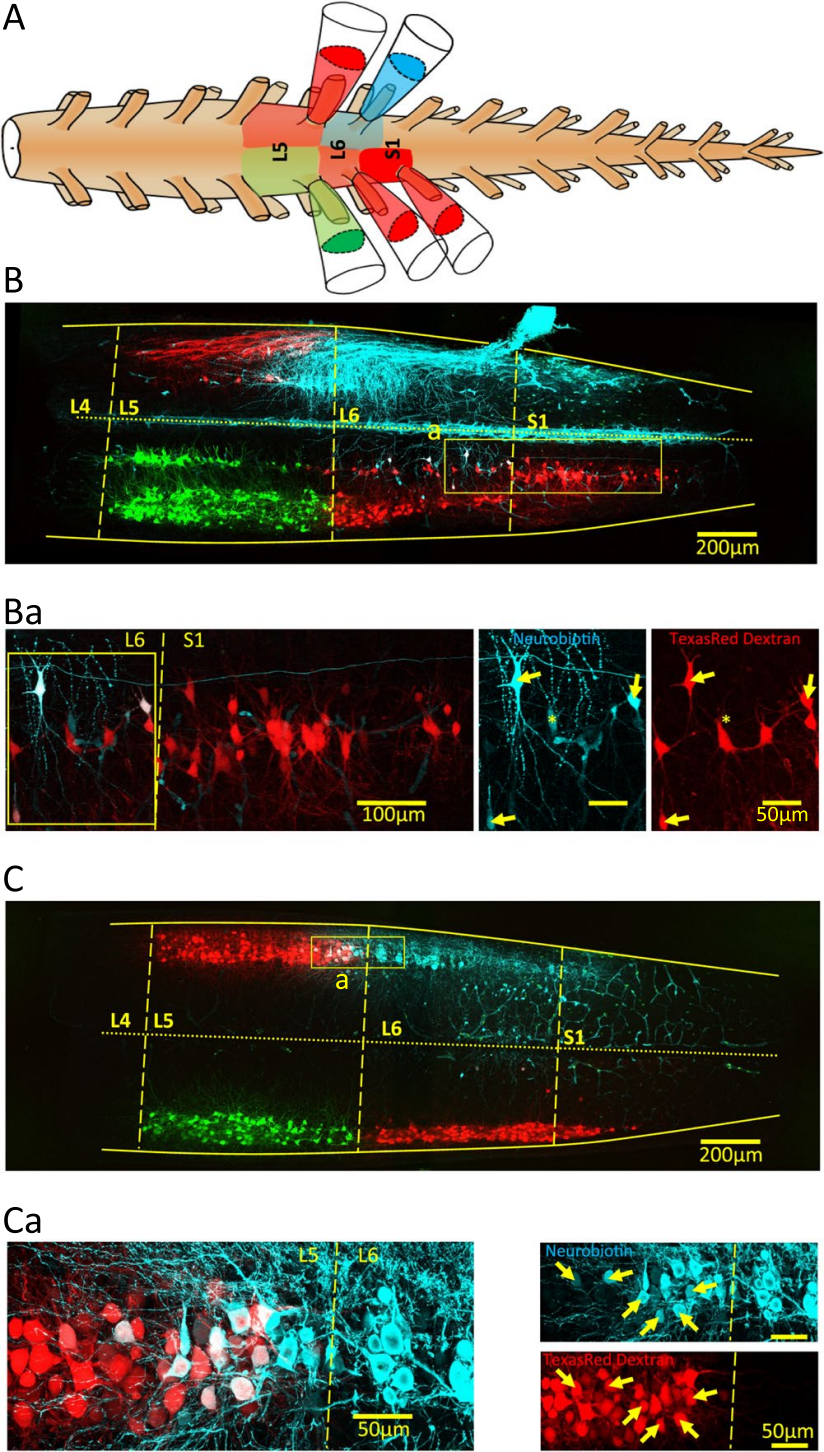
Coronal sections of the lower lumbar cord showing contralateral and inter-segmental dye-coupling. **(A**) Experimental arrangement. The left L6 ventral root was loaded with Neurobiotin (blue) while the right L5 (Fluorescein dextran; green), left L5, right L6 and S1 ventral roots (Texas Red Dextran; red) were filled with a high molecular weight dye. (**B**,**C**) Micrographs showing two contiguous z-stack projections (total of 200 µm from the ventral surface of the spinal cord). The segmental boundaries are defined by the dextran fills or by the exit point of the ventral root. (Ba) Expanded view of the cord contralateral to the Neurobiotin fill as delineated by the yellow rectangle in panel B. The yellow arrows in the righthand panels identify double labeled motoneurons and the asterisk marks a neuron only labeled from the contralateral Neurobiotin fill. Neurobiotin from the L6 ventral root labels motoneurons in the caudal part of the L5 segment. (Ca) Expanded image of the region outlined by the yellow rectangle in panel C, showing cells labeled with Neurobiotin from the L6 ventral root and also labeled with Texas Red Dextran from the L5 ventral root.

Motoneurons within the same pool have been shown to be coupled with gap junctions in the neonatal period^14–16^ and since pools span segments, we predicted that motoneurons should be dye-coupled across segment boundaries. To test this idea, one side of the cord was loaded with two different dextran (Fig. 3A–C, L5 green and L6-S1 red, dashed yellow line for the segment boundaries, which were defined by the dextran fills or by the rostral and caudal exit points of the ventral root). This also allowed us to verify that the neurons send their axons out through a single root and do not branch into adjacent roots. On the contralateral side, dextran and Neurobiotin (Fig. 3A, L5 red dextran, L6 cyan Neurobiotin) were used to assess whether there was any dye-coupling between the adjacent, ipsilateral segments. Figure 3B,C show two contiguous z-stack projections from ventral to caudal (100 µm each) encompassing segments L4 to S2. While there was no co-labeling of the dextran backfilled neurons in adjacent segments, co-labeling with dextran and Neurobiotin was observed, and as previously discussed, labeling contralateral to the neurobiotin fill was also seen (Fig. 3B). On the side of the L6 Neurobiotin fill, co-labeling was observed in dextran filled neurons located in the caudal ~150–200 µm of the L5 segment and in the rostral part of the S1 segment (Fig. 3B). Furthermore, Neurobiotin labeling was extensive in the caudal regions of the L5 segment, filling neurons and their dendrites (Fig. 3Ca, left panel).

**Figure 4:**
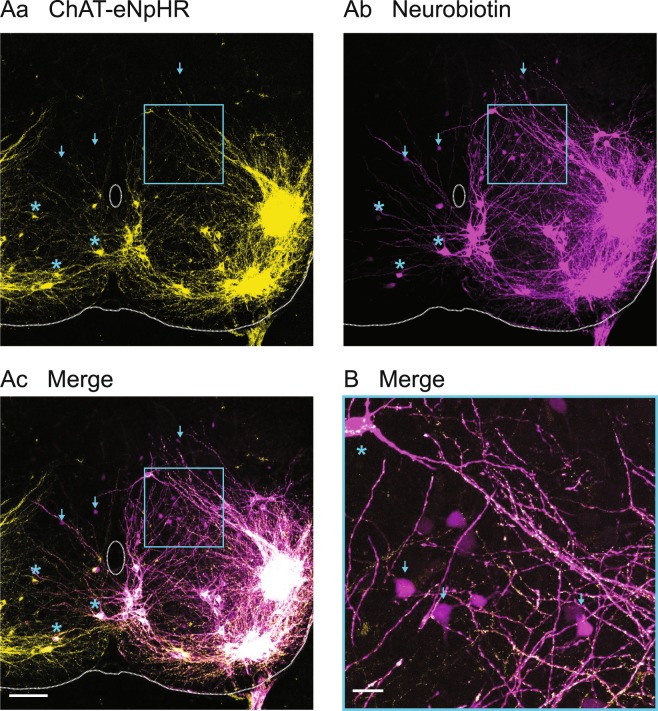
Putative interneurons are dye-coupled to motoneurons. (**A**) Z-stack projection of a 10X image of a 60 μm transverse section from a P3 ChAT-eNpHR mouse spinal cord showing the expression of halorhodopsin in ChAT-positive neurons in yellow (Aa), the Neurobiotin fill of the L6 ventral root in magenta (Ab), and the merged image (Ac). The arrows identify ChAT-negative neurons (putative interneurons) labeled with neurobiotin. The asterisks identify contralaterally labeled ChAT-positive neurons. (**B**) Z-stack projection of the 40X merged image of ChAT-halorhodopsin and Neurobiotin labeling from the region defined by the blue rectangle in A1–A3. Several ChAT-negative, Neurobiotin labeled, putative interneurons are shown (arrows) together with a double labeled cell (asterisks).

We also observed, but did not quantify, Neurobiotin-labeled neurons that were ChAT-negative in ChAT-Arch or ChAT-eNpHR mouse spinal cords and were located in regions of the cord that do not host motoneurons or parasympathetic preganglionic neurons. We presume that these cells are interneurons. We found them on the contralateral and ipsilateral sides of the fill, with a greater frequency of occurrence ipsilateral to the fill, mostly in laminae V to VII (Fig. 4A,B)

## Discussion

We have shown that prolonged (16–24 hours) application of Neurobiotin to the L6 ventral root labels ipsilateral motoneurons and parasympathetic preganglionic neurons as expected, and results in dye transfer to contralateral motoneurons and ipsilateral interneurons. Motoneuronal labeling outside of the backfilled segment and interneuronal labeling was never observed when the much larger dextran-conjugated dyes were used for the back-fill. The advantage of the method is that dye transfer from many neurons can be achieved, which maximizes the chance of detecting coupling particularly if the connections are sparse.

In neonatal rodents, hindlimb motoneurons belonging to the same pool, and to a lesser extent functional synergists, are coupled by dye-permeable, gap junctions^5,15–18^ and this coupling disappears or loses functionality after the first 2 neonatal weeks^14,19^. In the present study, we confirmed these results by showing that motoneurons were coupled across the L5/L6 segmental boundary following dye application to the L6 ventral root. Motoneurons in the L6 segment innervate several limb muscles (including the plantar foot muscles, gluteal muscles, hamstring muscles, and crural muscles) as well as the pelvic muscles. Some of the L6 motoneurons are sexually dimorphic, are more numerous in males and reside in two groups: the dorsolateral (DLN) and dorsomedial (DMN; also known as the spinal nucleus of the bulbocavernosus) motor nuclei. A previous study in the adult male rat used intracellular injection of single motoneurons in fixed slices to show that dye coupling is prominent in the sexually dimorphic BMN and DLN motor nuclei^12^. Interestingly, the authors report that even in adulthood motoneurons innervating flexor digitorum brevis (a non-sexually dimorphic muscle) show dye-coupling although less frequently than the sexually dimorphic motoneurons. The dye-coupled cells were not identified, and it was assumed some were motoneurons because of their size and location while other smaller cells were presumed to be interneurons. In addition, they showed that injections of the DMN motoneurons resulted in both ipsi- and contralateral labeling. The coupling was assumed to be mediated through gap junctions because it was reduced by the gap junction blocker oleamide. Our results largely confirmed the results of this earlier study, with the advantages that we could provisionally identify the coupled cells as motoneurons or interneurons and our experiments were performed in live tissue without the potential confound of fixation. We used both male and female mice in our experiments, suggesting that the dye-coupling is not restricted to the sexually dimorphic motoneurons of males. The L6 ventral root also contains axons of parasympathetic preganglionic neurons that were also filled with Neurobiotin. Although some preganglionic neurons have been shown to be electrically coupled and dye-coupled^20–22^, there is no evidence that they are coupled contralaterally or ipsilaterally to motoneurons or to interneurons.

Neurobiotin is small enough to pass through gap junctions^4,6^, although dye-coupling may underestimate the extent of coupling when compared to dual intracellular recordings^23^. Consistent with the idea that dye-coupling indicates the existence of gap junctions, it has been shown that the gap junction protein connexin-36 is densely expressed on sexually dimorphic motoneurons in the adult and neonatal mouse^24^. In our study, we never encountered either dye-filled contralateral motoneurons or putative interneurons when the back-fill was performed using high molecular weight dextran-conjugated dyes that do not traverse gap junctions. One caveat is that dye-coupling between neurons that are not connected by gap junctions has been reported^25^. In the mormyrid fish, dye-coupling was detected between retrogradely labeled, large fusiform projection neurons and satellite granular cells in the electrosensory lobe. Although these cells lacked gap junctions, they were connected by adhaerentia-like appositions that the authors termed neurapses and which were argued to mediate the dye transfer.

Dye-coupling between contralateral motoneurons has not be documented in the developing spinal cord. The location of the contralaterally labeled neurons corresponds to the position of the ventromedial DMN which contains neurons innervating the bulbocavernosus muscle. This muscle is activated during copulatory behavior^26^ so that the bilateral coupling may ensure that the left and right parts of the muscle are activated synchronously.

Dye transfer was also observed between motoneurons and putative interneurons. We characterized these cells as interneurons because of their small size, their location outside the motor nuclei and the absence of staining for ChAT. If dye-transfer reflects gap junctions and electrical coupling, this is the first time that spinal interneurons and motoneurons  have been shown to be coupled in the mammalian spinal cord. Whether or not this type of coupling between dissimilar neurons types is unique to the sexually dimorphic motoneurons remains to be determined.

The mechanism that allows the dye to enter the cut axons is not known. Cut axons of crayfish and giant squid axons seal in a calcium-dependent manner within a few minutes to an hour^27^. If the cut ventral root axons in our experiments sealed within an hour it would render the prolonged filling unnecessary. To determine if dye-loading occurred beyond these times we applied a suction electrode to the cut axons of the ventral root (n = 2) and waited 3–4 hours before backfilling it with Texas Red Dextran (10,000 MW). After leaving the preparation overnight we confirmed after sectioning the cord that motoneurons were retrogradely labeled with the dye. This result indicates that either the cut axons do not seal over this time or the dyes enter the ventral root axons by an unknown mechanism. The possibility that the dye labels cells following leakage from the electrode is very unlikely. If the dye had leaked into the aCSF it would have been substantially diluted into the 200–500 mL perfusate and the labeling pattern would have resembled that seen when fluorogold is applied extracellularly^28^, which was never seen.

## Materials and Methods

### Animals

All experiments were done according to the NIH guidelines and approved by the Animal Care and Use Committee of National Institute of Neurological Disorders and Stroke (Animal protocol number 1267). Spinal cords were isolated from neonatal (P0-3) male and female wild type mice (Swiss Webster, Taconic Laboratory) or transgenic mice. To distinguish between motoneurons and interneurons, we used mice expressing either green (GFP) or red fluorescent protein (RFP) in motoneurons or in subsets of interneurons. We used the following transgenic lines obtained from Jackson Laboratories: Floxed archaerhodopsin-GFP (Arch, B6;129S-*Gt(ROSA)26Sor*^*tm35.1(CAG-aop3/GFP)Hze*^/J, stock# 012735), floxed halorhodopsin-EYFP (eNpHR, B6;129S-*Gt(ROSA)26Sor*^*tm39(CAG-hop/EYFP)Hze*^/J, stock# 014539), floxed EGFP (EGFP, *Gt(ROSA)26Sor*^*tm1.1(CAG-EGFP)Fsh*^/Mmjax, MMRRC stock# 32037-JAX), and floxed Ai9-tdT (Ai9, B6.Cg-*Gt(ROSA)26Sor*^*tm9(CAG-tdTomato)Hze*^/J, stock#007909). We crossed the Arch, eNpHR, EGFP lines with ChAT-Cre (B6;129S6-*Chat* ^*(tm1cre)Lowl*^/J, stock# 006410), the Ai9 lines with VGluT2-Cre (*Slc17a6*^*tm2(cre)Lowl*^/J, stock# 016963) and Gad67-EGFP lines (CB6-Tg(Gad1-EGFP)G42Zjh/J, stock#007677) to obtain animals in which the fluorescent proteins were expressed in cholinergic, glutamatergic, and glutamatergic and GABAergic neurons respectively. In addition, we crossed the floxed EGFP line with *Engrailed1*-Cre mice which were kindly gifted by Martyn Goulding.

### Dissection

Following decapitation and evisceration, the mouse torso with hindlimbs attached was placed in a dissection chamber and superfused with cold artificial cerebrospinal fluid (aCSF; concentration in mM: 128.35 NaCl, 4 KCl, 1.5 CaCl_2_.H2O, 1 MgSO_4_.7H_2_O, 0.58 NaH_2_PO_4_.H2O, 21 NaHCO_3_, 30 D-glucose). A ventral laminectomy was preformed and the spinal cord with attached dorsal and ventral roots was extracted and allowed to reach room temperature.

### Dye preparation and loading

The ventral roots of interest were placed in suction electrodes loaded with dyes as previously described^29^. A low molecular weight dye (Neurobiotin; Vector Labs, SP-1120; FW 322.8) was dissolved in aCSF (2 mg in 10 µL solution) and a high molecular weight dye (Cascade Blue Dextran (D1976), Fluorescein Dextran (D1820) or Texas Red Dextran (D1863), (ThermoFisher Scientific/Invitrogen, FW 10,000), was dissolved in normal aCSF (1 mg dye in 6 µL solution). In all experiments, the loading of the dye was done at room temperature for 16 to 24 hours.

### Processing and imaging

After loading of the dye (16–24 hours), the electrodes containing the dye were carefully withdrawn to avoid any spillage of the dye into the extracellular solution. The cord was then cut, keeping only the L5 to S1 segments, that were immediately immersed in 4% paraformaldehyde in Phosphate Buffered Saline (PBS) and kept in this solution overnight at 4 °C. Next, the segments were washed in PBS and embedded in agar (5% in PBS) and serially sectioned (60–100 µm; VT1000S, Leica Biosystems, Buffalo Grove, IL). For preparations that were backfilled with dextran only, the sections were then mounted onto slides and cover-slipped with a Glycerol:PBS solution (3:7). The sections labeled with Neurobiotin were collected sequentially and separated into wells (five to ten sections per well) to be processed with free floating immunohistochemistry. First, sections were blocked in 10% normal donkey serum (Chemicon, #S30) diluted in PBS-T (0.01 M PBS/0.1% Triton-X). In some preparations, an antibody against Choline Acetyltransferase (anti-ChAT) was used to label cholinergic cells, or an antibody against peripherin (anti-peripherin, which labels neurons with peripheral projections e.g. motoneurons, preganglionic parasympathetic neurons and sensory afferents), and in preparations that expressed GFP in the cell membrane or in the cytoplasm, an antibody against GFP (anti-GFP) was employed. We used a goat anti-ChAT antibody (AB144P, Millipore, Temecula, CA), chicken anti-peripherin (AB9282, Millipore, Temecula, CA), or rabbit anti-GFP (ab 6556, Abcam, Cambridge, MA), and incubated them (1:100, 1:1000, 1:1000, in PBS-T respectively) overnight at room temperature. We then washed out the antibody or antibodies with successive 10 minute PBS-T washes for an hour. We subsequently used fluorophore-conjugated secondary antibody (Jackson Immuno Research, FITC and or Cy5 depending on the preparation we used; 1:50 in PBS-T, 3Hrs) and streptavidin fluorophore-conjugated (Vector laboratories: DyLight 488 Streptavidin (#SA-5488), DyLight 649 Streptavidin (#SA-5649), or AMCA Avidin D (A-2008); 1:100 in PBS-T, 3 Hrs) accordingly. In preparations where no primary antibodies were used, only the streptavidin fluorophore-conjugated was added for 3 hours after the donkey serum. Finally, following a 60 minute wash with PBS, the sections were mounted on slides and cover-slipped with a Glycerol:PBS solution (3:7). Sections were scanned using a confocal microscope (SP5, Leica Microsystems or a LSM510, Carl Zeiss, light imaging facility, NINDS) and processed with LAS AF software (Leica Microsystems) and/or ImageJ.

### Fluorescence issues

As discussed above, we tried different approaches to characterize the dye-coupled neurons. When using antibodies (anti-ChAT or anti-Peripherin) for this purpose, the fluorescence on the side of the fill, particularly in putative motoneurons, appeared to be quenched by the presence of Neurobiotin (data not shown). We therefore tried mouse lines that expressed fluorescent proteins in cholinergic neurons, or glutamatergic and/or inhibitory interneurons. However, for all these transgenic mice, we noticed that the fluorescence rapidly faded after mounting the slices on slides, making it impossible to scan all the slides from a single experiment. We obtained the best results – the least fading - with lines in which the fluorescent protein was membrane bound (eNpHR, Arch) and when the sections were scanned immediately after mounting.

### Data analysis

To quantify the contralateral, dye-coupled neurons, we scanned the sections into z-stack images that were then rotated and cropped, using the central canal and the surrounding white matter as reference points, so that only the analyzed hemicord was visible. All images were then scaled to the same size (500 × 800 pixels). After outlining each filled neuron soma, the soma was pseudo-colored and a binary image was created containing only the marked neurons. We used a similar approach to define and quantify the location of dextran-filled motoneurons and parasympathetic neurons within the L6 segment ipsilateral to the fill. Because the labeling was so dense in these fills, individual motoneurons could not be separated, so their location was grouped, marked and considered as one area. For each data set (contralateral Neurobiotin fill; control ipsilateral dextran fill) all binary images were stacked. The probability of observing labeling (of individual neurons or groups of neurons) per section, was calculated by summing the value of the pixels (1 or 0) across all the sections for a given experiment and dividing by the number of sections. This probability was then color coded as indicated in the figure. To calculate the probability of observing a labeled pixel per experiment, we generated a binary map where the pixel was coded as 1 if it was labeled in any section, and 0 otherwise. These maps were then averaged across experiments and color-coded. All image operations were performed using FIJI (Fig. 1C for dextran control; 2D for contralateral Neurobiotin dye-coupled neurons).

## Data Availability

The data that support the findings of this study are available from the corresponding author upon reasonable request.

## References

[1] Baker MW, Macagno ER (2014). Control of neuronal morphology and connectivity: emerging developmental roles for gap junctional proteins. FEBS Lett.

[2] Belousov AB, Fontes JD (2013). Neuronal gap junctions: making and breaking connections during development and injury. Trends Neurosci.

[3] Connors BW (2017). Synchrony and so much more: Diverse roles for electrical synapses in neural circuits. Dev Neurobiol.

[4] Devor A, Yarom Y (2002). Electrotonic coupling in the inferior olivary nucleus revealed by simultaneous double patch recordings. J Neurophysiol.

[5] Mentis GZ, Diaz E, Moran LB, Navarrete R (2002). Increased incidence of gap junctional coupling between spinal motoneurones following transient blockade of NMDA receptors in neonatal rats. J Physiol-London.

[6] Vaney DI (1991). Many diverse types of retinal neurons show tracer coupling when injected with biocytin or Neurobiotin. Neurosci Lett.

[7] Huang Q, Zhou D, DiFiglia M (1992). Neurobiotin, a useful neuroanatomical tracer for *in vivo* anterograde, retrograde and transneuronal tract-tracing and for *in vitro* labeling of neurons. J Neurosci Methods.

[8] Bass AH, Marchaterre MA, Baker R (1994). Vocal-acoustic pathways in a teleost fish. J Neurosci.

[9] Herrick JL, Keifer J (1998). Central trigeminal and posterior eighth nerve projections in the turtle Chrysemys picta studied *in vitro*. Brain Behav Evol.

[10] Birinyi A, Straka H, Matesz C, Dieringer N (2001). Location of dye-coupled second order and of efferent vestibular neurons labeled from individual semicircular canal or otolith organs in the frog. Brain Res.

[11] Racz E, Bacskai T, Halasi G, Kovacs E, Matesz C (2006). Organization of dye-coupled cerebellar granule cells labeled from afferent vestibular and dorsal root fibers in the frog Rana esculenta. J Comp Neurol.

[12] Coleman AM, Sengelaub DR (2002). Patterns of dye coupling in lumbar motor nuclei of the rat. J Comp Neurol.

[13] Falgairolle, M., Blivis, D. & O’Donovan, M. J. In *Society for Neuroscience* Vol. 782. 18 (Neuroscience Meeting Planner, San Diego, CA, 2013).

[14] Chang Q, Gonzalez M, Pinter MJ, Balice-Gordon RJ (1999). Gap junctional coupling and patterns of connexin expression among neonatal rat lumbar spinal motor neurons. J Neurosci.

[15] Fulton BP, Miledi R, Takahashi T (1980). Electrical synapses between motoneurons in the spinal cord of the newborn rat. Proc R Soc Lond B Biol Sci.

[16] Walton KD, Navarrete R (1991). Postnatal changes in motoneurone electrotonic coupling studied in the *in vitro* rat lumbar spinal cord. J Physiol.

[17] Falgairolle, M., Puhl, J. G., Pujala, A., Liu, W. & O’Donovan, M. J. Motoneurons regulate the central pattern generator during drug-induced locomotor-like activity in the neonatal mouse. *Elife* **6**, 10.7554/eLife.26622 (2017). 10.7554/eLife.26622 PMC5550280 28671548

[18] Tresch MC, Kiehn O (2000). Motor coordination without action potentials in the mammalian spinal cord. Nat Neurosci.

[19] Personius KE, Chang Q, Mentis GZ, O’Donovan MJ, Balice-Gordon RJ (2007). Reduced gap junctional coupling leads to uncorrelated motor neuron firing and precocious neuromuscular synapse elimination. Proc Natl Acad Sci USA.

[20] Logan SD, Pickering AE, Gibson IC, Nolan MF, Spanswick D (1996). Electrotonic coupling between rat sympathetic preganglionic neurones *in vitro*. J Physiol.

[21] Nolan MF, Logan SD, Spanswick D (1999). Electrophysiological properties of electrical synapses between rat sympathetic preganglionic neurones *in vitro*. J Physiol.

[22] Shen E, Dun NJ (1990). Neonate rat sympathetic preganglionic neurons intracellularly labelled with lucifer yellow in thin spinal cord slices. J Auton Nerv Syst.

[23] Hinckley CA, Ziskind-Conhaim L (2006). Electrical coupling between locomotor-related excitatory interneurons in the mammalian spinal cord. J Neurosci.

[24] Bautista W, McCrea DA, Nagy JI (2014). Connexin36 Identified at Morphologically Mixed Chemical/Electrical Synapses on Trigeminal Motoneurons and at Primary Afferent Terminals on Spinal Cord Neurons in Adult Mouse and Rat. Neuroscience.

[25] Meek J, Kirchberg G, Grant K, von der Emde G (2004). Dye coupling without gap junctions suggests excitatory connections of gamma-aminobutyric acidergic neurons. J Comp Neurol.

[26] Miura T, Kondo Y, Akimoto M, Sakuma Y (2001). Electromyography of male rat perineal musculature during copulatory behavior. Urol Int.

[27] Eddleman CS, Bittner GD, Fishman HM (2000). Barrier permeability at cut axonal ends progressively decreases until an ionic seal is formed. Biophys J.

[28] Falgairolle M, O’Donovan MJ (2015). Pharmacological Investigation of Fluoro-Gold Entry into Spinal Neurons. PLoS One.

[29] Blivis, D. & O’Donovan, M. J. Retrograde loading of nerves, tracts, and spinal roots with fluorescent dyes. *J Vis Exp*, 10.3791/4008 (2012). 10.3791/4008 PMC3466642 22547001

